# Supplementary material for: The health impacts of a 4-month long community-wide COVID-19 lockdown: Findings from a prospective longitudinal study in the state of Victoria, Australia
Source: PLoS One. 2022 Apr 7;17(4):e0266650. doi: 10.1371/journal.pone.0266650 (PMC8989338; doi:10.1371/journal.pone.0266650)
Supplement: S1 Fig — (DOCX) [file pone.0266650.s001.docx]

**Supplementary Figure S1. Changes in determinants of health prior to, during (8 July – 27 October 2020), and after the community lockdown in Victoria, compared to the Rest of Australia. Data describe group percentages and 95% confidence intervals.**
