# Supplementary material for: The health impacts of a 4-month long community-wide COVID-19 lockdown: Findings from a prospective longitudinal study in the state of Victoria, Australia
Source: PLoS One. 2022 Apr 7;17(4):e0266650. doi: 10.1371/journal.pone.0266650 (PMC8989338; doi:10.1371/journal.pone.0266650)
Supplement: S1 Table — (DOCX) [file pone.0266650.s003.docx]

**Supplementary Table S1. Summary of restrictions leading to, during, and following the 2020 extended community lockdown in Victoria, Australia.**

| **Restrictions** | **Date** | **Location in Victoria (VIC)** |
| --- | --- | --- |
| Postcode lockdown | 30 Jun | 10 postcodes areas in Melbourne |
| Postcode lockdown | 4 Jul | 2 additional postcodes areas in Melbourne |
| Interstate border closed between New South Wales (NSW) and Victoria | 8 Jul | NSW-VIC border |
| **Stage 3 restrictions.**  Four reasons to leave home:   - Shopping for essential items - Caregiving - Exercise (1 hour) - Work (employers must support you to work from home if you can work from home) | 8 Jul | Metropolitan Melbourne and Mitchell shire |
| Schools return to flexible and remote learning for pupils in Prep to Year 10 (i.e. ages 5/6 to 15/16) | 20 Jul | Metropolitan Melbourne and Mitchell shire |
| Face coverings mandatory | 22 Jul | Metropolitan Melbourne and Mitchell Shire |
| Visitor limits. No hosting visitors at home or visiting people at their home | 30 Jul | Otway-Colac region (6 local government areas west of Melbourne) |
| **Stage 4 restrictions.**   - Curfew from 8pm to 5am - 5km distance limit from home. - 1-hour exercise limit - Maximum gathering of two. - Shopping limited to one person per household. - Weddings banned (from 5 Aug) - All onsite students learning from home (5 Aug) | 2 Aug | Metropolitan Melbourne |
| Face coverings mandatory | 2 Aug | VIC |
| **Stage 3 restrictions.**  Four reasons to leave home:   - Shopping for essential items - Caregiving - Exercise (1 hour) - Work (employers must support you to work from home if you can work from home) | 5 Aug | Regional Victoria (including Mitchell Shire) |
| **Easing restrictions:** |  |  |
| **Step 1:**  **Metro Melbourne:**  Curfew from 9pm to 5am  One permitted visitor (bubble) for people living alone and single parents  Exercise and social interaction (2 hours)  Increased gathering limits  Onsite learning staged return from Term 4 (PREP to Grade 2, VCE and VCAL 12 Oct)  **Step 2**  **Regional VIC:**  One permitted visitor (bubble) for people living alone  Gatherings limit: 5 people outdoors from 2 households  Outdoor public pools and playgrounds open  Religious services outdoors: limit 5 people and a faith leader  Onsite learning staged return from Term 4 (all students between 12-16 Oct) | 13 Sep | Metropolitan Melbourne  and  Regional Victoria |
| **Melbourne:**  5km limit to 25 km limit  Any reason allowed for leaving home  No time limit on exercise and social interaction  Outdoor sport settings reopen  Allied health professionals resume face-to-face care  Hairdressers reopen  Gatherings limit: 10 people from 2 households in outdoor public places  **Regional VIC:**  Libraries limit of 20 people indoors  Outdoor religious gatherings limit 20 people  Hospitality: 40 customers indoors, up to 70 outdoors | 18 Oct | Metropolitan Melbourne  and  Regional Victoria |
| **Regional VIC + Greater Shepparton**  Gyms and fitness spaces: up to 20 people, density 1 person per 8 square metres  Indoor pool limit: 20 people  Indoor sport begins for people aged 18 and under  Food courts open  Live music resumes (outdoor hospitality)  Religious celebrations: 20 people limit indoors, 50 people outdoors, and a faith leader | 27 Oct | Regional VIC and Greater Shepparton |
| **112 days of lockdown ends**  **Metro Melbourne**  Further relaxation on visitors  Any reason permitted to leave home  25km limit from home  Ring of steal in place (work permits required for crossing boundary)  ring of steal in place  Restaurants and pubs reopen: limit of 50, 20 indoors  Remaining retail opens  Workplaces reopen (“if you can work from home you must work from home” in place) | 27 Oct | Metropolitan Melbourne |
| **Step 3**  No restrictions on intrastate travel  Restrictions eased in Metropolitan Melbourne to be consistent with regional VIC  2 people visit per day (different households, different visits) | 8 Nov | VIC |
| 15 visitors each day  Outdoor gathering limit 50  Religious ceremonies limit 150 people indoors  Hospitality: density limit 1 person per 2 square metres, up to 50 customers. QR code record keeping mandatory. Larger venue cap of 150 people. | 22 Nov | VIC |
| Masked limited to: public transport, some retail and indoor shopping centres / stores, supermarkets.  30 people visitor limit  Outdoor gatherings limit 100  Density limit restaurants, café, pubs 1 person per square metre, no cap. QR code record keeping mandatory. | 6 Dec | VIC |
