# Supplementary material for: The health impacts of a 4-month long community-wide COVID-19 lockdown: Findings from a prospective longitudinal study in the state of Victoria, Australia
Source: PLoS One. 2022 Apr 7;17(4):e0266650. doi: 10.1371/journal.pone.0266650 (PMC8989338; doi:10.1371/journal.pone.0266650)
Supplement: S2 Table — (DOCX) [file pone.0266650.s004.docx]

**Supplementary Table S2. Comparisons of participants included in the analyses (Retention group) and those excluded (Attrition group).**

| **Group N (%)** | **Retention group** | **Attrition group** |
| --- | --- | --- |
|  | Completed 3 surveys at all relevant time-points (baseline, 3- and 6-month follow-up surveys). | Completed baseline survey, but did not complete at least one of the 3- or 6-month follow-up surveys |
| **Total** | **898 (100.0)** | **1705 (100.0)** |
| **Demographics** |  |  |
| ***Gender*** |  |  |
| Female | 475 (52.9) | 1139 (66.8) |
| Male | 421 (46.9) | 554 (32.5) |
| ***Age group*** |  |  |
| 18 to 24 years | 67 (7.5) | 178 (10.4) |
| 25 to 34 years | 123 (13.7) | 319 (18.7) |
| 35 to 44 years | 150 (16.7) | 334 (19.6) |
| 45 to 54 years | 218 (24.3) | 429 (25.2) |
| 55 to 64 years | 268 (29.8) | 384 (22.5) |
| 65 or more years | 72 (8.0) | 61 (19.6) |
| **Pre-existing health** |  |  |
| ***Anxiety*** |  |  |
| Yes | 106 (11.8) | 357 (20.9) |
| No | 792 (88.2) | 1348 (79.1) |
| ***Depression*** |  |  |
| Yes | 139 (15.5) | 353 (20.7) |
| No | 759 (84.5) | 1352 (79.3) |
| **Survey mode** |  |  |
| Online form | 186 (20.7) | 1037 (60.8) |
| Telephone interview | 712 (79.3) | 668 (39.2) |
