# Supplementary material for: The health impacts of a 4-month long community-wide COVID-19 lockdown: Findings from a prospective longitudinal study in the state of Victoria, Australia
Source: PLoS One. 2022 Apr 7;17(4):e0266650. doi: 10.1371/journal.pone.0266650 (PMC8989338; doi:10.1371/journal.pone.0266650)
Supplement: S3 Table — (DOCX) [file pone.0266650.s005.docx]

**Supplementary Table S3. Comparisons of participants residing in Victoria compared to participants in the Rest of Australia.**

| **Group N (%)** | **Exposed Group**  **(**State of Victoria) | **Comparison group**  (Rest of Australia) |
| --- | --- | --- |
| **Total** | **305 (100.0)** | **593 (100.0)** |
| **Demographics** |  |  |
| ***Gender*** |  |  |
| Female | 166 (54.4) | 306 (51.6) |
| Male | 139 (45.6) | 279 (47.0) |
| ***Age group*** |  |  |
| 18 to 24 years | 25 (8.2) | 42 (7.1) |
| 25 to 34 years | 48 (15.7) | 74 (12.5) |
| 35 to 44 years | 61 (20.0) | 86 (14.5) |
| 45 to 54 years | 75 (24.6) | 143 (24.1) |
| 55 to 64 years | 69 (22.6) | 198 (33.4) |
| 65 or more years | 27 (8.9) | 44 (7.4) |
| **Pre-existing health** |  |  |
| ***Anxiety*** |  |  |
| Yes | 34 (11.1) | 72 (12.1) |
| No | 271 (88.9) | 515 (86.8) |
| ***Depression*** |  |  |
| Yes | 50 (16.4) | 88 (14.8) |
| No | 255 (83.6) | 499 (84.1) |
| **Survey mode** |  |  |
| Online form | 62 (20.3) | 124 (20.9) |
| Telephone interview | 243 (79.7) | 463 (78.1) |
