# Supplementary material for: The health impacts of a 4-month long community-wide COVID-19 lockdown: Findings from a prospective longitudinal study in the state of Victoria, Australia
Source: PLoS One. 2022 Apr 7;17(4):e0266650. doi: 10.1371/journal.pone.0266650 (PMC8989338; doi:10.1371/journal.pone.0266650)
Supplement: S4 Table — (DOCX) [file pone.0266650.s006.docx]

**Supplementary Table S4. Impacts of the lockdown in Victoria on determinants of health including work, social interactions and finance.**

|  | **Adjusted Odds Ratios [95% Confidence Interval]** | | | | | |
| --- | --- | --- | --- | --- | --- | --- |
|  | **Work** | | **Social interactions** | | **Finance** | |
|  | ***Not working hours*** | ***Unemployment*** | ***Social isolation*** | ***Virtual interactions*** | ***Fewer financial resources*** | ***Financial stress*** |
| **The Lockdown Impact** |  |  |  |  |  |  |
| During lockdown | **2.00** [1.40, 2.87] | 1.17 [0.77, 1.77] | **3.73** [2.52, 5.53] | 1.33 [0.96, 1.85] | 1.15 [0.73, 1.79] | 0.83 [0.59, 1.16] |
| After lockdown | 1.08 [0.69, 1.68] | 1.13 [0.67, 1.91] | 0.75 [0.49, 1.15] | 1.15 [0.82, 1.64] | 1.28 [0.81, 2.01] | 0.74 [0.52, 1.07] |
| **Changes in health over location and time** |  |  |  |  |  |  |
| VIC * pre-lockdown | **2.83** [1.96, 4.09] | **1.75** [1.13, 2.71] | **2.20** [1.57, 3.08] | **2.10** [1.57, 2.82] | **1.85** [1.17, 2.92] | **1.76** [1.28, 2.42] |
| VIC * lockdown | **2.58** [1.74, 3.80] | **1.73** [1.11, 2.68] | **6.49** [4.68, 9.00] | **1.95** [1.45, 2.62] | 1.24 [0.76, 2.05] | 1.26 [0.90, 1.77] |
| VIC * post-lockdown | 1.14 [0.73, 1.78] | 1.27 [0.79, 2.04] | 1.16 [0.80, 1.68] | 1.31 [0.97, 1.75] | 1.19 [0.72, 1.98] | 0.95 [0.67, 1.35] |
| RoA * (pre-lockdown) | **2.68** [2.01, 3.58] | **1.56** [1.13, 2.16] | **1.43** [1.10, 1.85] | **1.86** [1.51, 2.28] | **1.98** [1.49, 2.64] | **1.37** [1.11, 1.70] |
| RoA * (lockdown) | 1.22 [0.95, 1.56] | **1.32** [1.03, 1.69] | 1.13 [0.88, 1.45] | **1.30** [1.05, 1.60] | 1.16 [0.85, 1.59] | 1.20 [0.97, 1.47] |
| RoA * (post-lockdown) | 1.00 (ref.) | 1.00 (ref.) | 1.00 (ref.) | 1.00 (ref.) | 1.00 (ref.) | 1.00 (ref.) |

Estimates with P < .05 shown in bold. VIC – the state of Victoria (i.e. lockdown location during 8 July – 27 October 2020). RoA – Rest of Australia. Models were adjusted for gender, age group and survey mode. *The Lockdown Impact* describes health differences of working-age Victorians to the Rest of Australia, controlling for health differences pre-lockdown.
