# Supplementary material for: The health impacts of a 4-month long community-wide COVID-19 lockdown: Findings from a prospective longitudinal study in the state of Victoria, Australia
Source: PLoS One. 2022 Apr 7;17(4):e0266650. doi: 10.1371/journal.pone.0266650 (PMC8989338; doi:10.1371/journal.pone.0266650)
Supplement: S1 Appendix — (DOCX) [file pone.0266650.s007.docx]

**Appendix S1. Media statements and Victorian Government publications on restrictions (corresponding to those listed in Supplemental Table S1).**

Andrews D. Statement From The Premier. [media release] (2020 July 7) [cited 2021 June 3]. Available from <https://www.premier.vic.gov.au/statement-premier-74>

Andrews D. Statement From The Premier. [media release] (2020 June 30) [cited 2021 June 3]. Available from <https://www.premier.vic.gov.au/statement-premier-72>

Andrews D. Statement From The Premier. [media release] (2020 July 4) [cited 2021 June 3]. Available from <https://www.premier.vic.gov.au/statement-premier-73>

Berejiklian G. Border closure to protect NSW. [media release] (2020 July 6) [cited 2021 June 3]. Available from <https://www.nsw.gov.au/media-releases/border-closure-to-protect-nsw>

Andrews D. Return To Flexible And Remote Learning. [media release] (2020 July 12) [cited 2021 June 3]. Available from <https://www.premier.vic.gov.au/return-flexible-and-remote-learning>

Andrews D. Face Coverings Mandatory For Melbourne And Mitchell Shire. [media release] (2020 July 19) [cited 2021 June 3]. Available from <https://www.premier.vic.gov.au/face-coverings-mandatory-melbourne-and-mitchell-shire>

Andrews D. Statement From The Premier. [media release] (2020 July 30) [cited 2021 June 3]. Available from <https://www.premier.vic.gov.au/statement-premier-75>

Andrews D. Statement On Changes To Melbourne’s Restrictions. [media release] (2020 August 2) [cited 2021 June 3]. Available from <https://www.premier.vic.gov.au/statement-changes-melbournes-restrictions>

Andrews D. Statement On Changes To Regional Restrictions. [media release] (2020 August 2) [cited 2021 June 3]. Available from <https://www.premier.vic.gov.au/statement-changes-regional-restrictions>

Andrews D. Statement From The Premier. [media release] (2020 September 6) [cited 2021 June 3]. Available from <https://www.premier.vic.gov.au/sites/default/files/2020-09/200906%20-%20Statement%20From%20The%20Premier.pdf>

Victoria State Government. Metropolitan Melbourne – Summary of easing of restrictions at 11:59pm on Sunday 18 October and Sunday 1 November. Oct 2020. <https://www.premier.vic.gov.au/sites/default/files/2020-10/201018_Metro_Melb_.pdf>

Victoria State Government. Regional Victoria – Summary of easing of restrictions at 11:59pm on Sunday 18 October and Sunday 1 November. Oct 2020. <https://www.premier.vic.gov.au/sites/default/files/2020-10/Regional%20VIC_Easing%20of%20Restrictions_.pdf>

Andrews D. Statement from the premier. [media release] (2020 October 18) [cited 2021 June 3]. Available from <https://www.premier.vic.gov.au/statement-premier-77>

Victoria State Government. Regional Victoria - Summary of further easing in the Third Step. Oct 2020. <https://www.premier.vic.gov.au/sites/default/files/2020-10/Regional%20VIC%20Easing%20Restrictions%2025-10%20V3_converted.pdf>

Victoria State Government. Metro Melbourne - Summary of the Third Step and further easing of restrictions. Oct 2020. <https://www.premier.vic.gov.au/sites/default/files/2020-10/201026%20-%20Metro%20Melb%20Easing%20Restrictions.pdf>

Victoria State Government. Summary of statewide restrictions for the Third Step and Last Step of Victoria’s roadmap to reopening. 2020 Nov. <https://www.premier.vic.gov.au/sites/default/files/2020-11/201108%20-%20Third%20Steps.pdf>

Victoria State Government. Summary of Last Step Restrictions from 11.59pm On 22 November 2020. 2020 Nov. <https://www.premier.vic.gov.au/sites/default/files/2020-11/221120%20-%20Last%20Step%20restrictions%20.pdf>

Victoria State Government. COVIDSafe Summer – How we work in Victoria. 2020 Dec 6. <https://www.premier.vic.gov.au/sites/default/files/2020-12/201206%20-%20COVIDSafe%20Summer%20-%20How%20we%20work.pdf>

Victoria State Government. COVIDSafe Summer – How we live in Victoria. 2020 Dec 6. <https://www.premier.vic.gov.au/sites/default/files/2020-12/201206%20-%20COVIDSafe%20Summer%20-%20How%20we%20live.pdf>
