# Supplementary material for: The health impacts of a 4-month long community-wide COVID-19 lockdown: Findings from a prospective longitudinal study in the state of Victoria, Australia
Source: PLoS One. 2022 Apr 7;17(4):e0266650. doi: 10.1371/journal.pone.0266650 (PMC8989338; doi:10.1371/journal.pone.0266650)
Supplement: Attachment — Submitted filename: Responses to reviewer comments_2022march_sub.docx [file pone.0266650.s009.docx]

**Responses to reviewer comments**

**Manuscript ID:** PONE-D-21-24391

**Title:** The health impacts of a 4-month long community-wide COVID-19 lockdown: Findings from a prospective longitudinal study in the state of Victoria, Australia.

We thank the reviewers for their consideration of this paper and comments. Our responses are listed in the table below.

| **Reviewer comment** | **Author response** |
| --- | --- |
| ***Reviewer 2:***  The authors acknowledge that their methodology does not allow the capture of data on the most relevant possible mental health impact — post-traumatic stress disorder (PTSD).  This condition has a clear temporal element: onset of symptoms is often delayed until some time after a traumatising event. There is popular media discussion indicating mental health practitioners and the community itself are reckoning with potentially traumatic effects of lockdown in combination with alarm caused by the pandemic itself (e.g. ABC 2021; Coslett 2021; Sarner 2021; Watson 2021). Yet this paper repeatedly claims to demonstrate that mental health impacts did not persist following the conclusion of lockdown, a conclusion framed by the research questions as posed on lines 83-87 of the manuscript.  The authors used instruments (K-6, SF-12) that are not validated for the detection of PTSD. Although they measure symptoms that may accompany PTSD, these symptoms may have taken time to emerge following the cessation of lockdown. The study does not include data from any subsequent time points that could be used to assess whether there have been delayed effects from lockdown. The paper does not acknowledge the limited scope of its exploration of mental health until line 385: ‘Whilst our findings describe a recovery in aspects of mental health such as symptoms of anxiety and psychological distress…’  That rather more circumspect framing should be used throughout the entire paper, instead of referring to ‘mental health’ more generally.   REFERENCES  ABC 2021 https://www.abc.net.au/radio/melbourne/programs/theconversationhour/the-conversation-hour/13362804  Coslett 2021 https://www.theguardian.com/commentisfree/2021/dec/07/covid-ptsd-cases-mental-health-crisis-early-intervention-trauma-uk  Sarner 2021 https://www.theguardian.com/lifeandstyle/2021/apr/14/brain-fog-how-trauma-uncertainty-and-isolation-have-affected-our-minds-and-memory  Watson 2021 https://www.abc.net.au/everyday/lockdown-is-harder-than-before-trauma-specialist-explains-why/100252974 | The study objectives do not propose to measure post-traumatic stress disorder (PTSD) but rather it is designed to measure mental health generally – Mental Health as a person’s emotional, cognitive and intellectual status. It could be considered positive or negative at a given time.  Mental illness, or psychological disorders such as PTSD, is a different construct which can affect thinking, feeling, mood, behaviour and ability to function over time – we don’t propose to describe this outcome in our study objectives.  There is a difference between mental health and mental illness/conditions/disorders, although these can often be used interchangeably.  We claim to describe mental health using the SF-12 score (on a numeric scale) – an approach which is appropriate for this construct, validated, and well-defined in the Methods section.  We also describe a measure of overall physical health - analogously this does not describe specific types of physical impairments, or types of injury for example.  The reviewer states that the methodology does not directly capture post-traumatic stress disorders, yet the reviewer also describes that this is already acknowledged in the study limitations of the paper, citing part of the following sentence:  From Line 385:  “Whilst our findings describe a recovery in aspects of mental health such as symptoms of anxiety and psychological distress, mental disorders such as post-traumatic stress disorder may re-emerge over longer periods of time [33], or upon repeated exposure to lockdowns or crises.”  We do not propose to make claims about PTSD and we also acknowledge the limitation of the measures used in not being able to provide information about specific mental illnesses such as PTSD, Depression or Anxiety.  No changes to the text were made. |
| ***Reviewer 1 and Reviewer 4:***  All comments have been addressed. | N/A. |
